# Reply to Maxwell, A. Comment on “Miyamoto et al. Laryngopharyngeal Mucosal Injury Due to Nasogastric Tube Insertion during Cardiopulmonary Resuscitation: A Retrospective Cohort Study. *J. Clin. Med.* 2024, *13*, 261”

**DOI:** 10.3390/jcm13123448

**Published:** 2024-06-13

**Authors:** Kazuyuki Miyamoto, Hiromi Takayasu, Shino Katsuki, Atsuo Maeda, Keisuke Suzuki, Motoyasu Nakamura, Noriko Hida, Takehiko Sambe, Masaharu Yagi, Jun Sasaki, Munetaka Hayashi, Kenji Dohi

**Affiliations:** 1 Department of Emergency, Critical Care and Disaster Medicine, School of Medicine, Showa University, Hatanodai, Shinagawa-ku, Tokyo 1428666, Japan; rinomizuki@yahoo.co.jp (H.T.); katsukishinok@gmail.com (S.K.); atsuo@med.showa-u.ac.jp (A.M.); k.s.07202251@gmail.com (K.S.); motoyasu@med.showa-u.ac.jp (M.N.); masaharuy0130@gmail.com (M.Y.); jun-sa@da2.so-net.ne.jp (J.S.); munetaka@med.showa-u.ac.jp (M.H.); kdop@med.showa-u.ac.jp (K.D.); 2 Department of Emergency, Critical Care and Disaster Medicine, Showa University Fujigaoka Hospital, Fujigaoka Aoba-ku, Yokohama City 2278501, Japan; 3 Department of Clinical Pharmacy, Division of Clinical Research and Development, School of Pharmacy, Showa University, Kita-karasuyama, Setagaya-ku, Tokyo 1578577, Japan; n.hida@med.showa-u.ac.jp; 4 Department of Pharmacology, Clinical Pharmacology, School of Medicine, Showa University, Hatanodai, Shinagawa-ku, Tokyo 1428666, Japan; t-sambe@med.showa-u.ac.jp

We are grateful for the insightful comments provided by the author [1] on our recently published article [2]. Their analysis offers a valuable critique of the complexities associated with nasogastric tube (NGT) insertion in clinical practice. Regarding the criticisms that the authors raised for the described NGT insertion during Cardiopulmonary Resuscitation (CPR), we would like to address some comments.

With rapid technological advances, videoscopes have come to be widely used in emergency care, anesthesia, and surgery [2,3]. A video laryngoscope (VLS) enables safe and rapid tracheal intubation compared to previous methods.

Our study identified a case of NGT misplacement within the trachea. An examination of our records revealed a standard insertion time of 73 seconds and two attempts, signifying an uncomplicated procedure. In contrast, three patients who experienced unintentional tracheal advancement during blind insertion required 8–12 attempts, exceeding the median of 8 attempts observed in the difficult insertion group. As astutely noted by Maxwell, these patients may have experienced initial tracheal advancement, prompting repeated insertion efforts due to perceived resistance. It is well known that misplacement of an NGT in the respiratory tract induces a high risk of iatrogenic morbidity and mortality. In light of these findings, video laryngoscopy (VLS) emerges as a safe and time-efficient method for NGT insertion, eliminating the requirement for superfluous confirmatory tests like chest x-rays. However, the current impediment lies in cost, as VLS technology, while demonstrably cheaper in the past, remains less universally available. The frequency with which NGT advances to the trachea is not well understood for anatomical reasons. Thus, we believe it is important to clarify the frequency during CPR.

This scenario may be subject to change in the near future. An analogous situation can be observed with central venous catheter placement, which traditionally carried a risk of severe complications due to blind insertion [4]. The advent of ultrasound technology has revolutionized the process, leading to dramatic safety improvements through the routine use of ultrasound guidance [5]. Following a similar trajectory, the increased accessibility and affordability of VLS technology could pave the way for the widespread adoption of VLS-guided NGT insertion. As Maxwell aptly observes, intubation training with VLS on airway mannequins is already a common practice among emergency physicians, anesthesiologists, and intensive care physicians. This existing expertise could facilitate the rapid implementation of VLS training for NGT insertion.

The focus of our study was on patients receiving CPR, and it did not address potential hemodynamic changes associated with VLS use. Further research is warranted to explore the application of VLS-guided NGT insertion in patients with a broader spectrum of medical conditions. Additionally, Maxwell highlights the challenges faced by patients who are unable to utilize VLS, such as those with limited mouth opening or those in a prone position. A potential alternative could be nasolaryngoscopy-guided NGT insertion, while acknowledging the increased difficulty due to the limitations in laryngeal visualization compared to VLS. Indeed, Boston [6] reported the usefulness of the portable nasolaryngoscope and plain suture with the assistance of resident. However, there is still much debate about the best way to insert a gastric tube. We firmly believe that further research is essential to establish the full potential of VLS-guided NGT insertion.
